# How Often Are Hospitalised Children Physically Restrained During Painful and Stressful Procedures?

**DOI:** 10.1111/jocn.70068

**Published:** 2025-08-11

**Authors:** Danton Matheus de Souza, Lucy Bray, Vanderlei Amadeu Rocha, Edmara Bazoni Soares Maia, Aline Santa Cruz Belela Anacleto, Lisabelle Mariano Rossato

**Affiliations:** ^1^ Department of Maternal, Child, and Psychiatric Nursing, School of Nursing University of São Paulo. São Paulo Sao Paulo Brazil; ^2^ Child Health Literacy, Faculty of Health and Social Care Edge Hill University Ormskirk UK; ^3^ Department of Pediatric Nursing, Paulista School of Nursing Federal University of São Paulo Sao Paulo Brazil

**Keywords:** child advocacy, hospitalised child, pain management, painful procedures, physical restraint, psychological stress

## Abstract

**Aim:**

To analyse the incidence of physical restraint use during painful and stressful procedures in hospitalised children, as well as the factors associated with its use.

**Design:**

Observational, longitudinal and prospective study.

**Methods:**

Children aged between 28 days and 10 years in a public hospital in Brazil were each observed undergoing clinical procedures over a 6‐h period. Data were collected on demographics, observed pain using validated measures, stress behaviours, and the use of physical restraint. Descriptive and inferential analyses were performed. National ethical guidelines were strictly followed.

**Results:**

1210 procedures were observed on 75 children, including 351 painful and 859 stressful procedures. Physical restraint was used in 270 (22.3%) procedures; of these, 131 (48.5%) were painful procedures and 139 (51.5%) were stressful procedures. In stressful procedures, at least one stress‐related behaviour was observed before the initiation of physical restraint. Factors associated with increased use of physical restraint during painful procedures were younger children, with higher levels of care dependency, higher pain scores during procedures, and those who underwent intravenous medication administration, airway suctioning, tube insertion, and fixation changes. In stressful procedures, the factors associated with higher use of physical restraint were younger children, hospitalisation due to respiratory conditions, those who underwent physical examinations, inhaled medication, and nasal lavage; and the child's expression of stress behaviour before the procedure starts. Predictors of physical restraint included morning period, younger age group, male or female sex, and transfer from the Intensive Care Unit.

**Conclusion:**

A high incidence of physical restraints was observed across multiple painful and stressful procedures performed within a 6‐h period, associated with variables related to both the child's characteristics and the procedures.

**Implications for the Profession and/or Patient Care:**

This study aims to encourage reconsideration of the frequent use of physical restraint in paediatric procedures, calling for a reframing of its application as an unquestioned practice toward an approach that prioritises protecting and respecting a child as a subject with needs, rights, and desires.

**Reporting Method:**

Strengthening the Reporting of Observational studies in Epidemiology (STROBE).

**Summary**

- What problem did the study address?
  ○ Physical restraint in painful and/or stressful procedures is recommended only as a last resort, such as in emergencies. However, in paediatric practice, its use is often unquestioned and widespread, leading to negative impacts, violating rights, disregarding children's needs, and posing ethical challenges to clinical practice. Despite this, its epidemiology remains underexplored in the literature, particularly in developing countries such as Brazil.
- What were the main findings?
  ○ Our study advances evidence of the use of restraint within paediatric practice by conducting observations of practice instead of relying on self‐reports of the incidence of physical restraints. The study demonstrates a high incidence (22.3%) of restraint use in both painful procedures and non‐invasive stressful procedures, along with their associated factors and predictors.
- Where and on whom will the research have an impact?
  ○ We hope this study will have an impact on healthcare services, particularly by encouraging healthcare professionals to reflect on their practices and reconsider their role as protectors and promoters of child rights and care. We also hope it will serve as a starting point for discussions among policymakers, fostering an understanding that the frequent use of physical restraint needs to be reconsidered. Finally, we encourage more researchers to engage in studies on this phenomenon.
- No patient or public contribution
- What does this paper contribute to the wider global clinical community?
  ○ Our study expands the international knowledge on physical restraint, particularly by describing its observed use in a public hospital in a developing country. Examining the incidence and nature of physical restraint highlights the tensions that can exist between children's rights and the completion of clinical tasks.

## 1 Introduction

Children, understood as individuals between 28 days and under 10 years of age (World Health Organization 2018), are subjects of the present, with rights and needs. Current legislation stipulates that children are entitled to the protection and respect of their physical and mental health, the right to be free from any form of mistreatment or pain, access to accurate and age‐appropriate health information, and the assurance that their views and expressions will be appropriately acknowledged and taken into consideration in matters affecting them (Conselho Nacional dos Direitos da Criança e do Adolescente 1995). In addition to these rights, children have needs that must be recognised whilst receiving care such as the need for protection, respect for their individuality, developmentally appropriate experiences, and stable, supportive communities (Veríssimo 2017). The recognition of children's rights and needs has supported care philosophies such as Child‐Centered Care, which places the child at the core of healthcare, emphasising the importance of communication, respect for their preferences, and participation in decision‐making (Carter et al. 2024).

Despite significant advancements, certain settings, such as hospitals, continue to face tensions and challenges in respecting and addressing children's rights and needs. How children's rights and needs are respected can be influenced by their stage of development, in the case of infants, between 28 days and 2 years old, their lack of verbal communication often leads to their views and expressions not being sought or taken seriously, with limited visibility given to their feelings, desires, and needs. Among preschoolers, aged 3–6 years, exclusion from care is still observed, with the child positioned merely as a recipient of what healthcare professionals (HCPs) perceive as the best care. Finally, school‐aged children, between 6 and 10 years old, are often not heard, with their needs being dismissed and their rights invalidated (Claridge and Powell 2023; Kleye et al. 2021; Eull et al. 2023).

In Brazil, a study conducted with 30 hospitalised school‐aged children found that they reported HCPs did not communicate directly with them, but only with their families, leaving them without the opportunity to express their questions and making them feel shy. Notably, this situation becomes even more pronounced during clinical procedures performed during hospitalisation (Abe et al. 2025). This experience is consistent with findings from other studies conducted with children (Claridge and Powell 2023; Kleye et al. 2021; Eull et al. 2023), which reveal that, in addition to the lack of communication, HCPs can disregard their wishes and needs by using physical restraints to complete the procedure (Abe et al. 2025; Jang et al. 2024; Bray et al. 2019).

Painful and stressful procedures are frequently identified by children as the most distressing component of the hospitalisation experience (Claridge and Powell 2023; Kleye et al. 2021). Pain and/or stress can trigger emotional reactions such as fear, anxiety, and anger, reduce satisfaction with care, and lead to feelings of helplessness and hopelessness, which can have lasting effects on children's health experiences, sensitivity, learning and development (Eull et al. 2023; Alotaibi et al. 2018). In Canada, a study involving 86 hospitalised children found that 92% underwent at least one documented painful procedure within a 24‐h period during their hospital stay (Wilding et al. 2022). While there are no data on the incidence of stressful procedures, it is likely that the number is even higher. Notably, the reduction of pain and stress during procedures has been identified as a research priority in paediatric care for this decade (Modanloo et al. 2023). To advance this agenda, one critical area of focus is identifying factors that influence children's experiences of pain and stress during procedures (Lavigne et al. 2017), with one such factor being the use of physical restraint.

## 2 Background

Physical restraint is defined as any action used to obstruct a child's movement, often involving the use of force on the child's body while the child expresses signs of resistance (Silva et al. 2024). Its use is only endorsed for emergency situations, where significant harm may occur and definitions often highlight its use during procedures should only be as a last resort; and even then, for the least restrictive option to be used for the shortest time possible (Forsner et al. 2023; Lombart et al. 2020).

However, evidence suggests that physical restraint continues to be frequently used within paediatric practice internationally (Bray et al. 2018), although the reported use of physical restraint in the literature varies. In studies reporting its incidence, estimates ranged from 9% to 11% (Lombart et al. 2020) and HCPs in a cross‐sectional study with 872 participants indicated that 48% frequently used physical restraint on children (Bray et al. 2018). However, these numbers are predominantly based on self‐report data. The difficulty in measuring and reporting incidence may stem from the perception of physical restraint as an inherent part of paediatric care, accepted, unquestioned, and invisible in the practice of clinical procedures (Silva et al. 2024; Ostberg et al. 2024). There also remains a degree of confusion and uncertainty about what constitutes physical restraint, both within practice and the literature on restraint within paediatric practice, with terms and definitions being used interchangeably (Silva et al. 2024).

HCPs justify the use of physical restraint during procedures as linked to ensuring the child's safety, preventing injury from stress‐related behaviours or interference with the procedure; children not having the understanding required to undergo a procedure without holding, taking into account the parents' preferences; and due to workload pressures and time constraints, feeling obligated to perform the procedure quickly, using the physical restraint and viewing it as a ‘necessary evil’. Despite an increase in papers and studies focusing on the use of restraint within paediatric practice, there remain notable gaps in the evidence (Silva et al. 2024; Forsner et al. 2023; Lombart et al. 2020; Bray et al. 2018, 2015; Ostberg et al. 2024).

Firstly, the evidence is heavily reliant on self‐report data, and there are very few observational studies which have been conducted which focus on holding and restraint within paediatric practice (Bray et al. 2016). A recent scoping review (Silva et al. 2024) and narrative review (Bray et al. 2015) showed a predominance of qualitative studies focusing on the experience of those involved in the procedure; most studies were conducted in developed countries, with an emphasis on Europe.

Secondly, the evidence focuses on parental and professional views of restraint, with few papers attending to children's first‐hand perspectives. In Sweden, a study conducted with 12 children and adolescents regarding their experience with physical restraint during procedures indicated that they felt threatened; experiencing fear, panic, anger, and a desire to act aggressively in an attempt to escape the feeling of being trapped; with a sense of invisibility to HCPs as they were not heard, assuming a passive role with feelings of helplessness; and an increase in pain and stress during procedures. In the long term, the memory of physical restraint remained with deep scars, shaping their reactions to new procedures (Forsner et al. 2023). This research demonstrates the short‐ and longer‐term harm which can be caused using physical restraint. The current limitations in the evidence highlight the need for further exploration and robust investigation, especially in different social, cultural, and economic contexts.

The research question underpinning this study was: What is the incidence of physical restraint in hospitalised children undergoing painful and/or stressful procedures?

## 3 The Study

The aim of this study was to analyse the incidence of physical restraint use during painful and stressful procedures in hospitalised children, as well as the factors associated with its use.

## 4 Methods

### 4.1 Design Study

An observational, longitudinal, prospective quantitative study was conducted between April and June 2024. The study was considered longitudinal as the same child was observed over a six‐hour period, with multiple measurements during this time (Hulley et al. 2015).

### 4.2 Study Setting and Sampling

The study was conducted in a public secondary‐level hospital in the state of São Paulo, Brazil, specifically in the Paediatric Inpatient Unit. This service provides care for children aged 28 days to 10 years. The unit has five rooms, each with four beds, and five individual isolation beds, but with a maximum capacity of 15 children. We chose this follow‐up period considering that 6 h correspond to a typical HCPs shift, during which the same team is responsible for the child's care. This allowed us to observe a morning shift (between 7 a.m. and 1 p.m.) or afternoon shift (between 1 p.m. and 7 p.m.) period with a clear beginning, middle, and end of the planning and execution of care.

This article discusses a subset of a larger project that analysed the total number of painful and stressful procedures in hospitalised children. We began with a pilot study involving 20 children who were observed for 6 h. A sample size calculation was performed based on the results of the larger study, using the number of stressful procedures due to its greater variability (standard deviation [SD] = 4.42), with a margin of error of one and a 95% confidence interval, resulting in a minimum sample size of 75 children observed for 6 h. It is important to note that no changes to the study design were necessary following the pilot phase, and the children who participated in this pilot stage were also included in the final sample.

### 4.3 Inclusion and Exclusion Criteria

Inclusion criteria were children aged between 28 days and 10 years, who were hospitalised on the unit. Exclusion criteria were children awaiting transfer to other units or healthcare services, those with a planned discharge, and those who had previously participated in the study during the same hospitalisation. These criteria were established to reduce the likelihood of not being able to complete the full 6‐h observation due to transfers or discharges of the children, and to minimise selection bias by including a child who had previously participated.

In this study, painful procedures were defined as those that invade the body's integrity, causing or potentially causing tissue damage, involving the removal or introduction of materials into the airway, digestive system and urinary tract. Stressful procedures were defined as those that are non‐invasive but have the potential to cause physical or emotional harm (Carbajal et al. 2008). It is worth noting that all procedures were non‐urgent, and the children were clinically stable.

### 4.4 Instrument

Data were collected on the child's demographics, painful and/or stressful procedures experienced, pain and/or stress measurement, and the use of physical restraint. Table 1 indicates the variables collected for each child. The child's age was assessed as a continuous numerical variable and also analysed by developmental age groups, categorised as follows: infants (between 28 days and 2 years), preschoolers (3–6 years), and school‐aged children (6–10 years). The level of care dependency was determined by a paediatric care classification scale, which assesses aspects of care such as activity, monitoring, oxygenation, medications, skin care, mobilisation, and family support, and classified children in minimal care, intermediate, high dependency, semi‐intensive and intensive (Dini and Guirardello 2014). Specialties were classified based on previous speciality categories (Carvalho et al. 2022): respiratory, infectious, surgical, orthopaedic, neurological, nephrological, haematological, genetic and metabolic.

**TABLE 1:** Variables collected in this study.

Characterisation data	Age Gender Race (categorised as white and non‐white—yellow, indigenous, mixed, and black) Comorbidities Total length of stay, until the point of observation Admission route (Paediatric emergency or intensive care unit) Level of dependency Specialties
Painful procedures	Tourniquet for blood draw Punctures: arterial, capillary, lumbar, and venous Airway suctioning Insertion of tubes (nasogastric or bladder catheterisation) Placement of non‐invasive ventilation devices Physiotherapy, including respiratory and/or motor exercises Dressing changes Manipulation of drains Fixation and device removal Administration of intravenous medication
Pain score	Neonatal Infant Pain Scale‐ NIPS Face, Legs, Activity, Cry and Consolability‐ FLACC Numeric Verbal Scale
Stressful procedures	Oral or inhaled medication administration Manipulation of gastric, enteral, or bladder catheters (administration of medications; maintenance of device patency and/or hygiene of tubes and catheters) Placement of ventilatory devices Bedside radiological examinations Vital sign assessment Nasal lavage Physical examination Hygiene procedures
Stressful behaviours	Crying Agitation Irritability Resistance Refusal to undergo the procedures (verbal and non‐verbal)

Painful and stressful procedures were defined based on the definitions above (Carbajal et al. 2008). Although painful procedures can also cause stress, and stressful procedures may become painful depending on the professional's approach, in this study we categorised each procedure under only one category, based on its main characteristics. Specifically for painful procedures, pain scores were measured by the researcher using the scales: Neonatal Infant Pain Scale (NIPS) for children between 28 days and 2 months, with scores ranging from 0 to 7; Face, Legs, Activity, Cry and Consolability (FLACC) scale for children between 2 months and under 7 years, including those with neuropathy, with scores ranging from 0 to 10; and Numeric Verbal Scale for children over 7 years, with scores ranging from 0 to 10 (Mencía et al. 2022). At the end, an equivalence calculation was performed, transforming the NIPS score into the FLACC scale, allowing for a single score for all procedures (Table 1).

For the stressful procedures, stress behaviours such as crying, agitation, irritability, resistance, and refusal to undergo the procedures were observed and noted on a proforma; children could exhibit one or more of these behaviours during the same procedure. Finally, based on the definition of physical restraint provided in the background, it was observed whether the children experienced restraint (Yes/No) during the procedures (Table 1).

### 4.5 Data Collection

Children to be observed were selected through simple random sampling, using a lottery method to determine which rooms would be included in data collection. The Paediatric Inpatient Unit has a capacity of 15 beds, distributed across shared rooms and single occupancy rooms. Upon arriving in the field and assessing which children could be included in the study, the researcher conducted a random selection among shared and isolation beds, considering a minimum of two eligible children to be invited to participate in the study.

After selecting one of the groups, the researcher requested the parent's authorisation for their child's participation, which was formalised through the signing of the Informed Consent Form. For school‐aged children over 6 years old, the invitation was directed to them, and if they agreed to participate, the Assent Form was signed in addition to parental consent.

**FIGURE 1:**
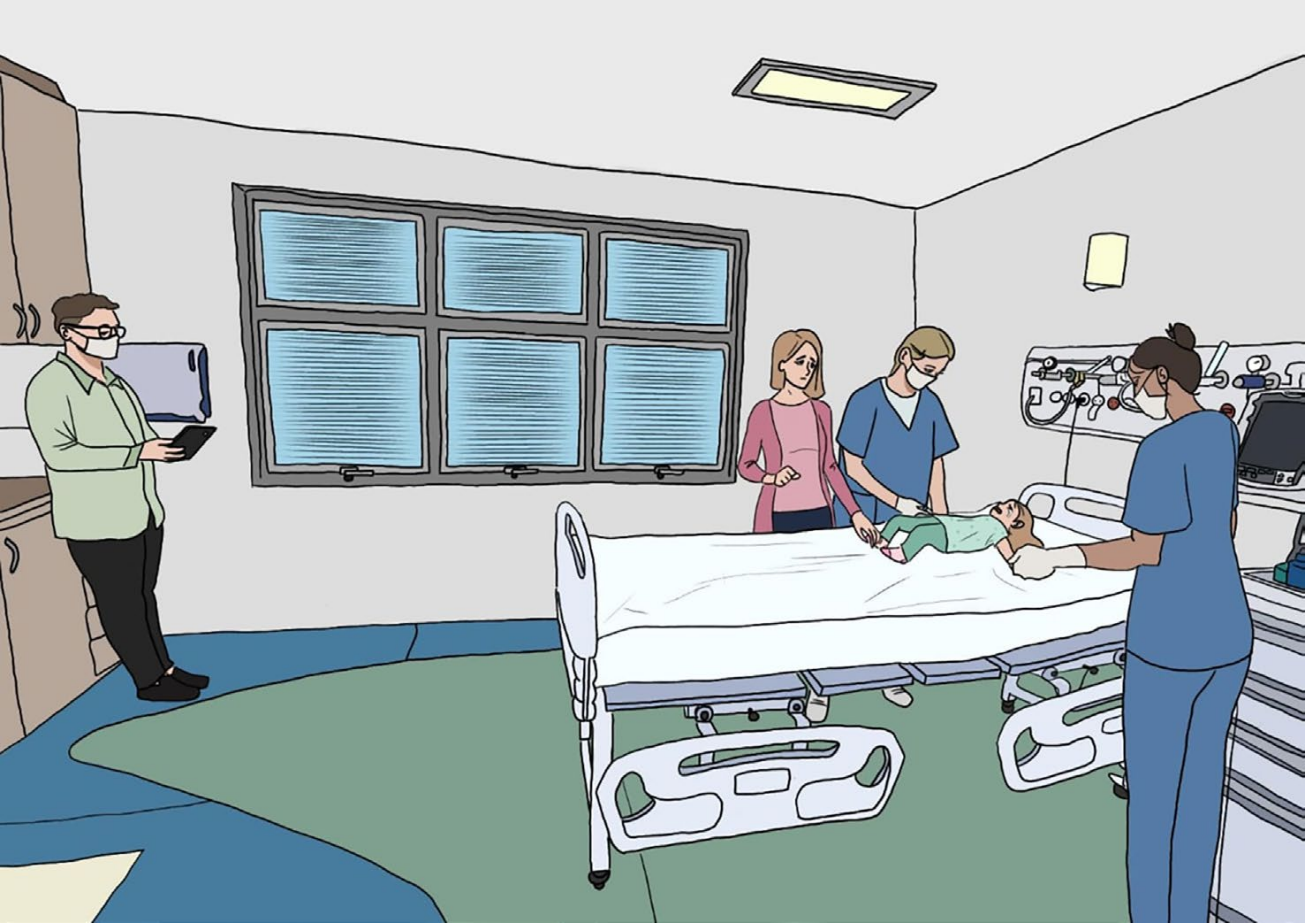
Representation of the researcher's presence at the bedside during data collection. [Colour figure can be viewed at wileyonlinelibrary.com]

Data collection was carried out by a single researcher—a nurse specialised in child and adolescent health, with a master's degree and currently a doctoral candidate in health sciences. This researcher remained in the paediatric inpatient unit (UIP), positioned near the child's bedside, while maintaining approximately one and a half metres to avoid influencing the procedure or the child's behaviour (Figure 1). To minimise the possibility of the Hawthorne effect—where HCPs might alter their behaviour due to the belief that they are being observed (Berkhout et al. 2022)—they were not informed about the study. Families and children were also asked not to disclose to the HCPs that the children were being observed.

### 4.6 Statistical Analysis

Data were tabulated and analysed using the R software through descriptive analysis, with percentiles, measures of central tendency (median and mean), and dispersion (SD and interquartile range—IQR); and inferential analysis using statistical tests. Initially, the Shapiro–Wilk test was used to determine the distribution of the variables. Subsequently, associations between the dependent variable (physical restraint) and the other independent variables were made using the Kruskal‐Wallis test, Mann–Whitney test, and binary logistic regression models. The confidence level was set at 95%.

### 4.7 Ethical Considerations

The study was approved by two ethics and research committees (proposing institution and research setting institution), in accordance with Brazilian legislation No. 466/2012 of the National Health Council. A data management plan was formulated and attached to the DMPTool platform. To guide the manuscript writing, we followed the recommendations of the Strengthening the Reporting of Observational Studies in Epidemiology (STROBE) instrument (Equator Network 2022).

## 5 Results

### 5.1 Characterisation of Participants and Procedures

Seventy‐five children were observed. The mean age was 2 years, with a predominance of infants (28 days to 2 years) (*n* = 55; 73.3%), followed by school‐age children (6–10 years) (*n* = 12; 16%) and preschoolers (3–6 years) (*n* = 8; 10.7%). Most participants were male (*n* = 46; 61.3%), non‐white (*n* = 38; 50.7%), and did not have underlying diseases (*n* = 64; 85.4%). The children had been hospitalised for an average of 107 h (SD: 377.8), mainly admitted from the paediatric emergency department (*n* = 50; 66.7%), with respiratory conditions (*n* = 51; 68%). Additionally, 83.7% (*n* = 62) had experienced at least one invasive procedure, with a peripheral venous catheter being the most common (*n* = 31; 41.3%). Regarding the level of care dependency, 48% (*n* = 36) were classified as requiring intermediate care, followed by 38.7% (*n* = 29) as high dependency, 12% (*n* = 9) as minimal care, and 1.3% (*n* = 1) as semi‐intensive care (Figure S1).

**FIGURE 2:**
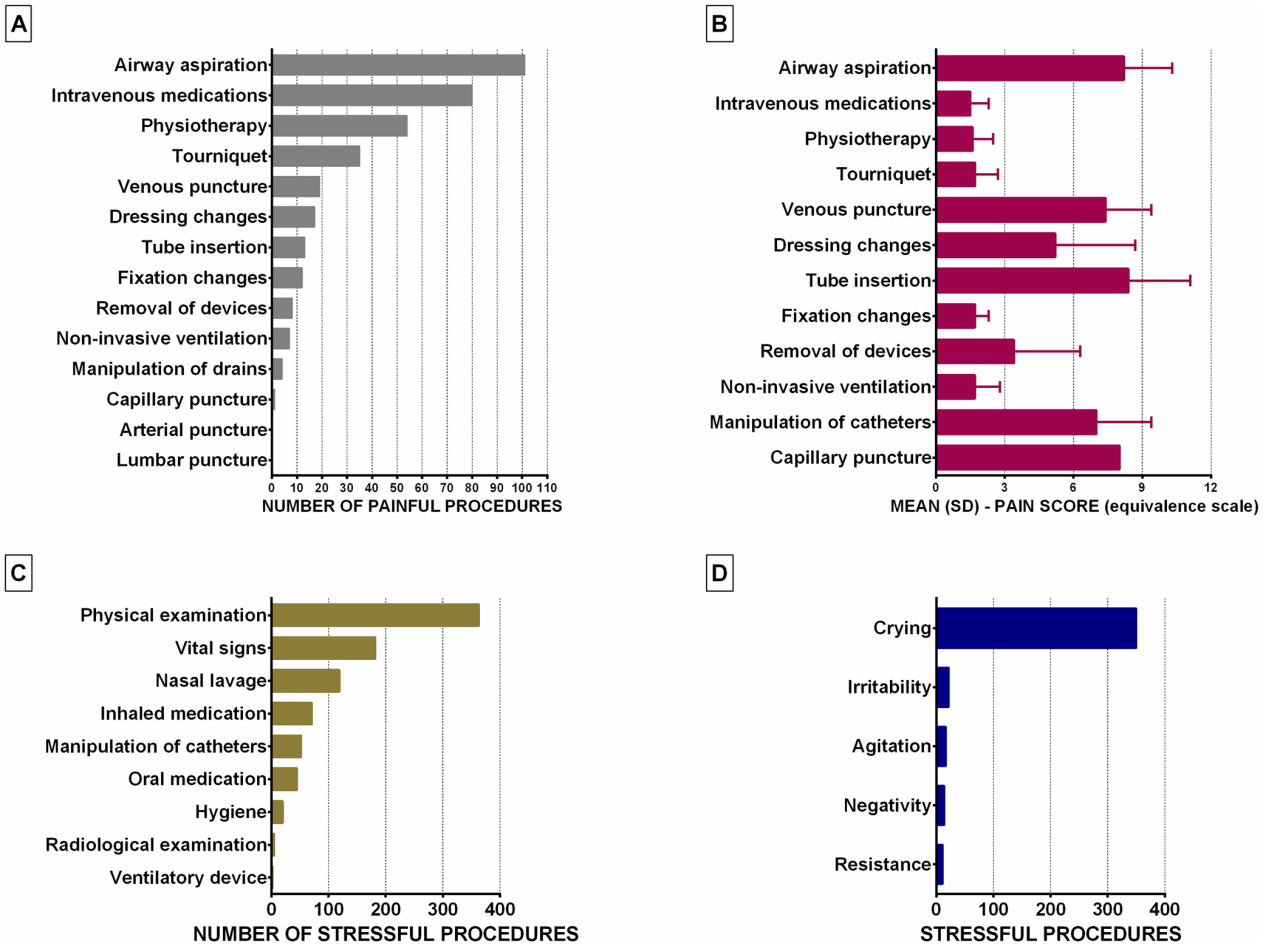
Characterisation of painful and stressful procedures. [Colour figure can be viewed at wileyonlinelibrary.com]

During the six‐hour observation period, 1210 procedures were recorded across all 75 children, with an average of 16 per child, ranging from a minimum of three to a maximum of 41 procedures. Of these, 351 were classified as painful procedures, with an average of four per child, ranging from 1 to 19. The most frequently performed painful procedures were airway suctioning (*n* = 101; 28.7%), intravenous medication administration (*n* = 80; 22.8%), and physiotherapy (*n* = 54; 15.4%) (Figure 2A). As shown in Figure 2B, pain assessments revealed that the most painful procedures were airway suctioning (mean: 8.2; SD: 2.1), nasogastric or urinary catheter insertion (mean: 8.4; SD: 2.7), and capillary puncture (mean: 8.0). Regarding stressful procedures, 859 were recorded, with an average of 11 per child, ranging from 2 to 22 procedures. The most frequently observed stressful procedures were physical examinations (*n* = 363; 42.2%), vital sign assessments (*n* = 182; 21.2%), and nasal lavage (*n* = 119; 13.8%) (Figure 2C). Among the stressful procedures, 414 were associated with at least one stress‐related behaviour, with crying being the most prevalent (84.5%) (Figure 2D). Finally, a family member was present with the child during all procedures (100%).

### 5.2 Characterisation of Physical Restraints in Procedures

Two hundred and seventy physical restraints were observed, with an average of three restraints per child within the six‐hour observation period. Of these, 131 physical restraints were applied during painful procedures, with an average of one per child. The most frequently observed restraints occurred during airway suctioning (*n* = 131), tourniquet application for venipuncture (*n* = 14), and tube insertions (*n* = 11). In stressful procedures, 139 physical restraints were observed, with an average of one per child. These were most applied during nasal lavage (*n* = 78), inhaled medication (*n* = 46), and oral medication (*n* = 11). In all cases of physical restraint during stressful procedures, children exhibited at least one stress‐related behaviour before, with crying being the most frequent (*n* = 124) (Table 2).

**TABLE 2:** Characterisation of physical restraints during procedures.

	Mean (SD)	Median (IQR)	95% CI
Physical restraints during procedures	3.6 (4.2)	3.0 (0–5.0)	2.86–4.82
Physical restraints during painful procedures	1.7 (2.5)	1.0 (0–3.0)	1.3–2.46
Physical restraints during stressful procedures	1.8 (2.3)	1.0 (0–3.0)	1.4–2.47

		Physical restraints	
		Yes	No
	*n*	*n* (%)	*n* (%)
Painful procedures	351.0	131.0 (37.3)	220.0 (62.7)
Airway aspiration	101.0	77.0 (76.2)	24.0 (23.8)
Intravenous medications	80.0	1.0 (1.2)	79.0 (98.8)
Physiotherapy	54.0	1.0 (1.8)	53.0 (98.2)
Tourniquet	35.0	14.0 (40.0)	21.0 (60.0)
Venous puncture	19.0	9.0 (47.4)	10.0 (52.6)
Dressing changes	17.0	7.0 (41.2)	10.0 (58.8)
Tube insertion (gastric or urinary catheter)	13.0	11.0 (84.6)	2.0 (15.4)
Fixation changes	12.0	7.0 (58.3)	5.0 (41.7)
Removal of devices	8.0	3.0 (37.5)	5.0 (62.5)
Non‐invasive ventilation	7.0	0	7.0 (100.0)
Manipulation of drains	4.0	1.0 (25.0)	3.0 (75.0)
Capillary puncture	1.0	0	1.0 (100.0)
Stressful procedures	859.0	139.0 (16.2)	720.0 (83.8)
Physical examination	363.0	2.0 (0.5)	361.0 (99.5)
Vital signs	182.0	2.0 (1.1)	180.0 (98.9)
Nasal lavagem	119.0	78.0 (65.5)	41.0 (34.4)
Inhaled medication	71.0	46.0 (64.8)	25.0 (35.2)
Manipulation of catheters	52.0	0	52.0 (100.0)
Oral medication	45.0	11.0 (24.4)	34.0 (75.6)
Hygiene	20.0	0	20.0 (100.0)
Radiological examination	5.0	0	5.0 (100.0)
Ventilatory device	2.0	0	2.0 (100.0)
Behaviours during stressful procedures	414.0	139.0 (33.6)	275.0 (66.4)
None	445.0	0	445.0 (100.0)
Crying	350.0	124.0 (35.4)	226.0 (64.6)
Irritability	22.0	2.0 (9.1)	20.0 (90.9)
Agitation	17.0	5.0 (29.4)	12.0 (70.6)
Negativity	14.0	2.0 (14.3)	12.0 (85.7)
Resistance	11.0	6.0 (54.5)	5.0 (45.5)

### 5.3 Factors Associated With Physical Restraint in Procedures

In painful procedures, younger age, higher dependency level, and procedure type were factors associated with the chance of being physically restrained. Higher rates of physical restraint were observed among infants (*p* < 0.001), children classified as requiring high‐dependency care (*p* < 0.001), and those undergoing airway suctioning (*p* < 0.001), tube insertion (*p* = 0.002), and dressing changes (*p* = 0.020). Children who were restrained during intravenous medication administration had a lower overall number of restraints (*p* = 0.016) (Table 3).

**TABLE 3:** Factors associated with the number of physical restraints in painful procedures.

	Number of physical restraints		
	Mean (SD)	Median (IQR)	*p*
Follow‐up period			
Morning	1.6 (2.5)	1.0 (0–2.0)	0.708^a^
Afternoon	1.9 (2.4)	1.0 (0–3.0)	
Age group			
Infants	2.1 (2.6)	2.0 (0–3.0)	**< 0.001** ^b^
Preschoolers	1.8 (2.3)	0 (0–4.0)	
School‐age	0	0	
Sex			
Male	1.8 (2.7)	1.0 (0–2.0)	0.649^a^
Female	1.7 (1.9)	1.0 (0–3.0)	
Skin colour			
White	2.2 (3.1)	2.0 (0–3.0)	0.366^a^
Non‐white	1.3 (1.5)	1.0 (0–2.0)	
Comorbidities			
No	1.8 (2.6)	1.0 (0–3.0)	0.327^a^
Yes	1.2 (1.7)	0 (0–2.5)	
Origin place			
Paediatric emergency room	1.5 (2.4)	1.0 (0–2.0)	0.086^a^
Intensive care unit	2.3 (2.5)	2.0 (0–4.0)	
Level of care dependency classification			
Minimal care	0.4 (0.7)	0 (0–1.0)	**< 0.001** ^b^
Intermediate care	1.0 (1.5)	0 (0–2.0)	
High dependency care	3.1 (3.2)	2.0 (1.0–4.0)	
Semi‐intensive care	0	0	
Speciality			
Infectious	1.2 (1.4)	0.5 (0–2.5)	0.131^b^
Respiratory	2.1 (2.7)	2.0 (0–3.0)	
Surgical	1.3 (2.1)	0 (0–2.5)	
Orthopaedic	0	0	
Neurological	1.2 (1.5)	1.0 (0–2.2)	
Haematological	0	0	
Genetic	0	0	
Airway aspiration			
No	0.6 (1.3)	0 (0)	**< 0.001** ^a^
Yes	2.5 (2.7)	2.0 (1.0–3.0)	
Intravenous medications			
No	1.9 (2.0)	2.0 (0–3.0)	**0.016** ^a^
Yes	1.4 (3.1)	0 (0–1.0)	
Physiotherapy			
No	1.5 (2.4)	0 (0–3.0)	0.226^a^
Yes	1.8 (2.5)	2.0 (0–2.0)	
Tourniquet			
No	1.4 (1.6)	1.0 (0–2.0)	0.282^a^
Yes	4.0 (5.3)	3.0 (0–5.0)	
Venous puncture			
No	1.4 (1.6)	1.0 (0–2.0)	0.282^a^
Yes	4.0 (5.3)	3.0 (0–5.0)	
Dressing changes			
No	1.8 (2.5)	1.0 (0–3.0)	0.466^a^
Yes	1.2 (1.8)	0 (0–2.0)	
Tube insertion			
No	1.4 (1.8)	1.0 (0–2.0)	**0.002** ^a^
Yes	6.2 (5.2)	3.0 (3.0–7.0)	
Fixation changes			
No	1.4 (2.2)	1.0 (0–2.0)	**0.020** ^a^
Yes	3.6 (3.2)	3.0 (1.2–5.0)	
Removal of devices			
No	1.7 (2.5)	1.0 (0–2.2)	0.594^a^
Yes	1.7 (1.6)	1.0 (0.5–3.0)	
Non‐invasive ventilation			
No	1.8 (2.5)	1.0 (0–3.0)	0.239^a^
Yes	1.0 (2.2)	0 (0)	
Manipulation of drains			
No	1.7 (2.5)	1.0 (0–3.0)	1.000^a^
Yes	1.5 (2.1)	1.5 (0.7–2.2)	

*Note:* Bold values denote statistical significance at the *p* < 0.05 level.

^a^ Wilcoxon‐Mann–Whitney test.

^b^ Kruskal‐Wallis test.

In stressful procedures, the factors associated with physical restraints were age, specialty, type of procedure, and behaviours. Infants (*p* = 0.002), children hospitalised due to respiratory conditions (*p* = 0.041), those undergoing inhalation medication administration (*p* < 0.001) and nasal lavage (*p* < 0.001), as well as those who exhibited stress‐related behaviours before the procedure (*p* < 0.001), particularly crying (*p* = 0.001), had a higher likelihood of being physically restrained. In contrast, children who were restrained during physical examinations experienced fewer instances of physical restraint in other procedures (*p* = 0.026) (Table 4).

**TABLE 4:** Factors associated with the number of physical restraints in stressful procedures.

	Number of physical restraints		
	Mean (SD)	Median (IQR)	*p*
Follow‐up period			
Morning	1.9 (2.6)	1.5 (0–3.0)	0.928^a^
Afternoon	1.7 (1.9)	1.0 (0–3.0)	
Age group			
Infants	2.3 (2.5)	2.0 (0–4.0)	**0.003** ^b^
Preschoolers	1.5 (1.9)	0.5 (0–2.8)	
School‐age	0.2 (0.5)	0 (0)	
Sex			
Male	2.1 (2.5)	2.0 (0–3.8)	0.397^a^
Female	1.5 (1.9)	0 (0–2.0)	
Skin colour			
White	2.1 (2.7)	2.0 (0–3.0)	0.653^a^
Non‐white	1.6 (1.8)	1.0 (0–3.0)	
Comorbidities			
No	1.9 (2.5)	1.0 (0–4.0)	0.787^a^
Yes	1.3 (1.1)	2.0 (0–2.0)	
Origin place			
Paediatric emergency room	1.9 (2.5)	1.0 (0–3.0)	0.948^a^
Intensive care unit	1.7 (1.9)	2.0 (0–3.0)	
Level of care dependency classification			
Minimal care	1.3 (1.6)	1.0 (0–2.0)	0.132^b^
Intermediate care	1.3 (1.8)	0 (0–2.3)	
High dependency care	2.7 (2.9)	2.0 (0–4.0)	
Semi‐intensive care	0	0 (0)	
Speciality			
Infectious	0.8 (0.9)	0.5 (0–1.8)	**0.007** ^b^
Respiratory	2.5 (2.5)	2.0 (0–4.0)	
Surgical	0.1 (0.4)	0 (0)	
Orthopaedic	0	0	
Neurological	1.0 (1.1)	1.0 (0–2.0)	
Haematological	0	0	
Genetic	0	0	
Physical examination			
No	5.5 (0.7)	5.5 (5.3–5.8)	**0.026** ^b^
Yes	1.7 (2.3)	1.0 (0–3.0)	
Vital signs			
No	2.0 (2.0)	2.0 (2.0–2.0)	0.732^a^
Yes	1.8 (2.3)	1.0 (0–3.0)	
Nasal lavage			
No	0.6 (1.1)	0 (0–1.0)	**< 0.001** ^a^
Yes	2.8 (2.6)	2.0 (0–4.0)	
Inhaled medication			
No	0.9 (1.3)	0 (0–2.0)	**< 0.001** ^a^
Yes	2.9 (2.8)	2.5 (0–5.0)	
Manipulation of catheters			
No	1.8 (2.4)	1.0 (0–3.0)	0.511^a^
Yes	2.1 (2.0)	2.0 (0–3.0)	
Oral medication			
No	1.6 (2.1)	0 (0–3.0)	0.062^a^
Yes	2.7 (2.9)	2.0 (1.5–3.0)	
Hygiene			
No	1.7 (2.4)	1.0 (0–2.8)	0.533^a^
Yes	2.1 (2.3)	2.0 (0–4.0)	
Radiological examination			
No	1.7 (1.9)	1.0 (0–3.0)	0.628^a^
Yes	3.6 (5.4)	2.0 (0–3.0)	
Ventilatory device			
No	1.8 (2.3)	1.0 (0–3.0)	0.944^a^
Yes	1.5 (2.1)	1.5 (0.8–2.3)	
**Stress behaviours**	2.2 (2.4)	2.0 (0–4.0)	**< 0.001** ^a^
Crying			
No	0.5 (1.1)	0 (0)	**0.001** ^a^
Yes	2.3 (2.4)	2.0 (0–4.0)	
Agitation			
No	1.9 (2.4)	1.5 (0–3.0)	0.762^a^
Yes	1.4 (1.6)	1.0 (0–2.0)	
Irritability			
No	1.6 (1.8)	1.0 (0–3.0)	0.170^a^
Yes	3.5 (4.2)	3.0 (0–6.0)	
Resistance			
No	1.8 (2.4)	1.0 (0–3.0)	0.694^a^
Yes	1.8 (1.4)	2.0 (1.0–2.0)	
Negativity			
No	1.9 (2.3)	1.5 (0–3.0)	0.359^a^
Yes	0.6 (1.1)	0 (0–1.0)	

*Note:* Bold values denote statistical significance at the *p* < 0.05 level.

^a^ Wilcoxon‐Mann–Whitney test.

^b^ Kruskal‐Wallis test.

Based on the analysis of each procedure, the following factors were identified as predictors for physical restraint: morning period; infants and school‐age; admission route from intensive care unit; and male or female sex (Table 5). In the morning period, children showed more restraints during painful procedures such as tourniquet (*p* = 0.039) and venipuncture (*p* = 0.042); in stressful procedures such as physical examination (*p* < 0.001) and in stress‐related behaviours during these procedures, such as irritability (*p* = 0.042). Infants showed a higher number of restraints in the painful procedure of airway aspiration (*p* < 0.001); while in stressful procedures, the number of behaviours (*p* = 0.034), crying (*p* < 0.001), and physical examination (*p* = 0.010) and nasal lavage (*p* = 0.006) were associated with a higher number of physical restraints. School‐age children showed more restraints during the painful procedure of intravenous medication (*p* = 0.003). Those hospitalised in the intensive care unit had a higher number of restraints during the painful procedure of dressing changes (*p* = 0.017) and in stressful procedures such as ventilatory device insertion (*p* = 0.046) and device removal (*p* = 0.025). Finally, males were associated with physical restraints during the stressful procedure of oral medication (*p* = 0.014), while females were associated with restraints during the painful procedures of tourniquet (*p* = 0.013), venipuncture (*p* = 0.014), and non‐invasive ventilation (*p* = 0.049).

**TABLE 5:** Predictors associated with the types of procedures and their physical restraints.

		Mean (SD)	Median (IQR)	*p*
**Follow‐up period (painful procedures)**				
Tourniquet	Morning	0.7 (1.9)	0 (0)	**0.039** ^a^
	Afternoon	0.1 (0.7)	0 (0)	
Venous puncture	Morning	0.4 (1.1)	0 (0)	**0.042** ^a^
	Afternoon	0.1 (0.5)	0 (0)	
**Follow‐up period (stressful procedures)**				
Physical examination	Morning	5.6 (1.6)	6.0 (4.0–6.0)	**< 0.001** ^a^
	Afternoon	3.8 (1.9)	4.0 (3.0–5.0)	
Irritability	Morning	0.4 (1.0)	0 (0)	**0.042** ^a^
	Afternoon	0.1 (0.6)	0 (0)	
**Age‐group (painful procedures)**				
Airway aspiration	Infants	1.7 (1.2)	2.0 (1.0–2.0)	**< 0.001** ^b^
	Preschoolers	1.2 (1.8)	0 (0–1.7)	
	School‐aged	0	0	
Intravenous medications	Infants	0.7 (1.7)	0 (0)	**0.003** ^b^
	Preschoolers	0.7 (1.2)	0 (0)	
	School‐aged	2.5 (2.0)	3.0 (0.7–4.0)	
**Age‐group (stressful procedures)**				
Physical examination	Infants	5.2 (1.8)	5.0 (4.0–6.0)	**0.010** ^b^
	Preschoolers	3.0 (2.0)	2.5 (2.0–4.0)	
	School‐aged	4.8 (1.6)	4.5 (3.7–6.0)	
Nasal lavagem	Infants	1.9 (1.7)	2.0 (0–3.0)	**0.006** ^b^
	Preschoolers	1.2 (1.6)	0 (0–2.0)	
	School‐aged	0.3 (0.7)	0 (0)	
Crying	Infants	6.0 (3.9)	6.0 (4.0–8.0)	**< 0.001** ^b^
	Preschoolers	3.0 (3.2)	2.0 (0.2–5,2)	
	School‐aged	0	0	
**Origin place (painful procedures)**				
Dressing changes	Paediatric emergency room	0.1 (0.2)	0 (0)	**0.017** ^a^
	Intensive care unit	0.5 (1.0)	0 (0–1.0)	
Removal of devices	Paediatric emergency room	0.1 (0.2)	0	**0.025** ^a^
	Intensive care unit	0.2 (0.5)	0	
**Origin place (stressful procedures)**				
Ventilatory device	Paediatric emergency room	0	0	**0.046** ^a^
	Intensive care unit	0.1 (0.3)	0	
Sex (painful procedures)				
Tourniquet	Male	0.2 (0.8)	0	**0.013** ^a^
	Female	0.9 (2.2)	0	
Venous puncture	Male	0.1 (0.5)	0	**0.014** ^a^
	Female	0.4 (1.2)	0	
Non‐invasive ventilation	Male	0 (0.1)	0	**0.049** ^a^
	Female	0.2 (0.5)	0	
**Sex (stressful procedures)**				
Oral medication	Male	0.9 (1.7)	0 (0–1.0)	**0.014** ^a^
	Female	0.1 (0.4)	0	

*Note:* Bold values denote statistical significance at the *p* < 0.05 level.

^a^ Wilcoxon‐Mann–Whitney test.

^b^ Kruskal‐Wallis test.

### 5.4 Probability of Physical Restraint in Procedures

Based on a logistic regression analysis, it was found that the likelihood of children being subjected to physical restraint during painful procedures (32.9%; 95% CI: 24.2%–43.1%) was higher than during stressful procedures (8.9%; 95% CI: 5.9%–13.1%) (*p* < 0.001). Specifically, age and pain score influenced the findings: for each additional year of age, the likelihood of restraint decreased by 25.6% (OR: 0.744; 95% CI: 0.674–0.821; *p* < 0.001), and for each one‐point increase on the pain equivalence scale, the likelihood of physical restraint during painful procedures increased by 2.3 times (OR: 2.344; 95% CI: 1875‐2,93; *p* < 0.001) (Table S1).

## 6 Discussion

This appears to be the first prospective study to systematically observe and document the use of physical restraint in paediatric patients during painful and stressful procedures. Physical restraint was used in 22.3% of the 1210 procedures observed, a rate that differs from estimates reported in the existing literature. A narrative review of 29 studies reported estimated restraint rates of 84% in radiology services, 71% in emergency departments, and 68% in intensive care units (Bray et al. 2015). However, those studies were conducted in settings where children were required to remain motionless during procedures and/or in high‐complexity healthcare environments. In contrast, our investigation was conducted in a low‐complexity setting with clinically stable children. This contrast may suggest that, despite the lower‐complexity context, the observed incidence of physical restraint was still notably high.

In the present study, the use of physical restraint was more frequent during painful procedures, with a 32.9% likelihood, increasing with higher pain scores. Restraint was most observed during airway suctioning, catheter insertions, and dressing changes. These findings align with a scoping review (Silva et al. 2024) that identified venipuncture, tube insertion, and anaesthetic induction as procedures frequently associated with restraint.

Similarly to previous studies, infants were more likely to be physically restrained than other age groups. HCPs have reported feeling more comfortable holding them and overriding their expressions of distress, which is concerning, as this age group seeks trust in their caregivers and environment, expressing emotions such as crying or smiling to communicate their feelings (Silva et al. 2024; Forsner et al. 2023; Ostberg et al. 2024; Bray et al. 2015; Merck and McElfresh 2014). However, as observed in our study, infants who cried and displayed a high number of stress behaviours were often ignored and were more likely to be restrained.

In stressful procedures, physical restraint was associated with at least one stress‐related behaviour, with crying being the most prominent. This supports existing literature, which relates emotional expressions like anger, resistance, discomfort, agitation, and refusal to increased use of physical restraint (Silva et al. 2024; Forsner et al. 2023; Lombart et al. 2020; Bray et al. 2016). A study in Sweden found that children previously subjected to restraint experienced anger and aggression, which strained their relationship with HCPs (Forsner et al. 2023). This is consistent with our findings, where high incidences of stress‐related behaviours during physical restraint may indicate that children are anticipating negative experiences and trying to communicate their reluctance to undergo them again.

Due to their developmental stage, children may exhibit stress‐related behaviours in response to situations they perceive as threatening, such as painful procedures (Forsner et al. 2023). These reactions are developmentally appropriate and should be respected as a valid form of expression. Children are not expected to remain silent during procedures; instead, the goal is for them to understand the purpose of the intervention and to be engaged in a manner that encourages cooperation, thereby reducing the need for physical restraint.

Children classified as highly dependent due to the need for more complex care were more frequently restrained during procedures, as were those transferred from the intensive care unit. This finding suggests that clinical severity was a factor associated with a higher frequency of restraints. A previous study reported that children in intensive care undergo an average of seven painful procedures per day (Baarslag et al. 2019), a number that may be even higher when stressful procedures are also considered. Given the elevated rates of physical restraint reported in this setting (Bray et al. 2015), it can be suggested that children's reactions to HCP are intensified by memories of past experiences, thereby contributing to the increased use of physical restraints during procedures.

Hospitalisation due to respiratory conditions, particularly among infants, was associated with the use of restraint during procedures. This finding may be attributed to the reduced ability of infants to expectorate secretions, thereby requiring more frequent interventions such as tracheal suctioning, nasal irrigation, and inhaled medications. Considering that respiratory conditions are the leading cause of hospitalisation in children, these findings suggest that this group may be more vulnerable to the consequences of unrelieved pain and stress during healthcare interventions (Souza et al. 2024).

The morning follow‐up period was associated with the number of physical restraints used in procedures such as tourniquet, venipuncture, and physical examination, as well as in children who exhibited stress‐related behaviours. In the co‐participating institution, a teaching hospital, the number of health sciences students is higher in the morning, leading to an increased number of procedures. This may have heightened the likelihood of stress and fear in children, resulting in greater use of restraints due to lower cooperation during procedures.

In parallel with the frequency of physical restraint use in children and the associated factors identified in this study, the number of procedures performed—ranging from 1 to 41 within 6‐h periods of observed care practice—can be considered alarming and unacceptably high. The continued pattern of practice observed—concerning the use of restraint, and the pain and stress associated with the performance of procedures without preparatory, distraction, or relief interventions—demands analysis from an ethical perspective. Children are inherently vulnerable, particularly when hospitalised. Given their rights, concern for their well‐being should be paramount, along with the prevention of harm (Carnevale and Manjavidze 2016). Children must be treated with respect, and the use of physical restraint should never be considered standard practice. It is necessary to reflect on possibilities for change, one of which is health advocacy.

Health advocacy, defined as the use of strategies to defend children by translating their rights into clinical practice (Conselho Nacional dos Direitos da Criança e do Adolescente 1995), can be a solution through the empowerment of children, families, and HCPs. For children, empowerment involves informed, respectful, and participatory practices, positioning them as active subjects in procedures (Silva et al. 2024; Alshammari et al. 2025). One example is the use of instructional therapeutic play to prepare children for procedures (de Souza et al. 2025). Another approach is the informed consent model, where children should be asked for authorisation before physical restraint, respecting their autonomy in decision‐making (Bray et al. 2018; Dalton and Doupnik 2024).

Another way to ensure advocacy for children and reduce physical restraint is using comfort positions, recommended for children over three years old. This approach allows the child to determine their position from available options, such as sitting, lying down, or being held up by a family member, providing a sense of control and autonomy (Kleye et al. 2021; Silva et al. 2024; Ostberg et al. 2024; Bray and Isupport 2021). For infants, facilitated tucking is an alternative. This technique stabilises the head and limbs, restricting movement without completely preventing it, and should begin three minutes before the procedure and continue afterwards (Neto et al. 2020).

Additionally, attention to the child during the procedure should be provided, especially for pain relief and promotion of distraction, using non‐pharmacological interventions. These may include breastfeeding, non‐nutritive sucking, play, parental support, holding, massage, and similar approaches (Guillari et al. 2024). It is possible that not all strategies will succeed, and physical restraint may be necessary during the procedure. In this case, it is valuable for the HCP to take the time to discuss with the child so that they can understand the process and reframe it, seeing it as the only alternative to promoting their well‐being.

HCPs should be empowered in advocating for children's rights. They must develop as leaders who prioritise childcare and pain relief as an obligation, establishing actions that focus on the child with an individualised approach. The nurse can stand out in this process, as they are the HCP who spend the most time with the child, empowering themselves and sharing this philosophy with others (Simons et al. 2020). Continuing education can be a valid strategy.

Our study is limited by the presence and observation of the researcher, which may have influenced the practice of the HCPs who may not have applied the number of physical restraints they typically use in daily clinical practice. The study was conducted in a single hospital, limiting the ability to compare and generalise the findings; it was also carried out in a clinical inpatient unit with stable children, which does not allow for generalisation to critically ill children. There is a prevalence of infants with respiratory issues but without underlying diseases, which restricts the possibility of more in‐depth analysis. No data were collected regarding the actions of the children's parents or other family members during the procedures, which limits the ability to determine whether they participated in the physical restraint. Furthermore, the data were analysed independently, rather than through a grouped‐variable approach.

For the future, we recommend new studies that aim to move in the opposite direction of the reality observed in our setting, questioning the use of physical restraints and viewing their use as an ethical dilemma and a violation of children's rights.

## 7 Conclusion

Over a six‐hour period, 1210 procedures were observed in children, with 351 being painful and 859 stressful. The incidence of physical restraint was 270, with an average of three per child, including 131 restraints in painful procedures and 139 in stressful ones. The probability of physical restraint occurrence was higher in painful procedures. Factors associated with higher rates of physical restraint during painful procedures were younger children, with higher levels of care dependency, higher pain scores, and the type of procedure. In stressful procedures, the associated factors were younger children, hospitalisation due to respiratory conditions, the type of procedure, and the child's expression of stress behaviour before the procedure starts. Predictors of physical restraint included morning period, age group, sex, and admission route.

## 8 Relevance to Clinical Practice

Our study advances the international knowledge frontier, focusing on physical restraints in children, occurring in a developing country with low investment in scientific research. We hope that its results can impact clinical practice, prompting HCPs to reflect on their practices, which still violate rights, ignore needs, and cause impacts on children; influence policymakers by highlighting the frequent use of physical restraint; and encourage researchers to engage in continuing studies on the phenomenon.

## Author Contributions

D.M.S. and L.M.R. worked on the conception and design of the study. D.M.S. worked on the analysis of data. D.M.S., L.B., V.A.R., E.B.S.M., A.S.C.B.A. and L.M.R. worked on the interpretation of data, writing, and critical review of the manuscript.

## Disclosure

The authors affirm that the methods used in the data analyses are suitably applied to their data within their study design and context; the statistical findings have been implemented and interpreted correctly.

## Ethics Statement

The Ethics and Research Committees of the School of Nursing at the University of São Paulo and the collaborating institution approved the study (Resolution No. 466/2012 of the National Health Council), ensuring compliance with the principles of the Declaration of Helsinki.

## Conflicts of Interest

The authors declare no conflicts of interest.

## Supporting information


**Data S1:** jocn70068‐sup‐0001‐Table_S1.docx.

**Data S2:** jocn70068‐sup‐0002‐Figure_S1.tif.

**Data S3:** jocn70068‐sup‐0003‐Strobe_Checklist.docx.

## Data Availability

The data that support the findings of this study are available from the corresponding author upon reasonable request.
