# Supplementary material for: How Often Are Hospitalised Children Physically Restrained During Painful and Stressful Procedures?
Source: J Clin Nurs. 2025 Aug 11;35(2):822–38. doi: 10.1111/jocn.70068 (PMC12779204; doi:10.1111/jocn.70068)
Supplement: Supplementary file 1 — Data S1: jocn70068‐sup‐0001‐Table_S1.docx. [file JOCN-35-822-s002.docx]

Table 1 – Probability of Receiving Physical Restraint During Procedures.

|  | **OR** | **CI 95%** | **p value*** |
| --- | --- | --- | --- |
| **Age (years)** | 0.744 | 0.674-0.821 | **<0.001** |
| **Pain score** | 2.344 | 1.875-2.930 | **<0.001** |
|  | **Probability** | **CI 95%** | **p value*** |
| **Type of procedure:** |  |  |  |
| Painful procedure | 32.9% | 24.2-43.1% | **<0.001** |
| Stressful procedure | 8.9% | 5.9-13.1% |  |

Legend: OR (Odds Ratio); 95% CI (95% Confidence Interval)

*Binomial logistic regression model.
